# Myc promotes glutaminolysis in human neuroblastoma through direct activation of glutaminase 2

**DOI:** 10.18632/oncotarget.5821

**Published:** 2015-10-19

**Authors:** Daibiao Xiao, Ping Ren, Hexiu Su, Ming Yue, Ruijuan Xiu, Yufeng Hu, Hudan Liu, Guoliang Qing

**Affiliations:** ^1^ School of Basic Medicine, Tongji Medical College, Huazhong University of Science & Technology, Wuhan 430030, China; ^2^ Department of Pharmacology, School of Pharmacy, Hubei University of Science & Technology, Xianning 437100, China; ^3^ School of Pharmacy, Tongji Medical College, Huazhong University of Science & Technology, Wuhan 430030, China; ^4^ Medical Research Institute, Wuhan University, Wuhan 430071, China

**Keywords:** neuroblastoma, N-Myc, glutaminase 2, glutamine, cancer metabolism

## Abstract

Deamidation of glutamine to glutamate by glutaminase 1 (GLS1, also called GLS) and GLS2 is an essential step in both glutaminolysis and glutathione (GSH) biosynthesis. However, mechanisms whereby cancer cells regulate glutamine catabolism remains largely unknown. We report here that N-Myc, an essential Myc family member, promotes conversion of glutamine to glutamate in *MYCN*-amplified neuroblastoma cells by directly activating GLS2, but not GLS1, transcription. Abrogation of GLS2 function profoundly inhibited glutaminolysis, which resulted in feedback inhibition of aerobic glycolysis likely due to thioredoxin-interacting protein (TXNIP) activation, dramatically decreasing cell proliferation and survival *in vitro* and *in vivo*. Moreover, elevated GLS2 expression is significantly elevated in *MYCN*-amplified neuroblastomas in comparison with non-amplified ones, correlating with unfavorable patient survival. In aggregate, these results reveal a novel mechanism deciphering context-dependent regulation of metabolic heterogeneities, uncovering a previously unsuspected link between Myc, GLS2 and tumor metabolism.

## INTRODUCTION

Neuroblastoma is one of the most frequent solid tumors detected in childhood. Risk factors indicative of poor prognosis include age >18 months at diagnosis, unfavorable histological grade and *MYCN* amplification [1]. Amplification of the *MYCN* gene, which occurs in approximately 25% of human neuroblastomas overall and 40% of high-risk cases, remains the most important and reliable oncogenic marker [1]. *MYCN* amplification is consistently associated with high levels of N-Myc protein, which contribute to aggressive phenotypes by regulating and/or cooperating with other oncogenic pathways [1, 2].

Central metabolic pathways and energy production differ between normal and malignant cells in their regulation and dynamics. Fast-growing tumor cells typically exhibit increased aerobic glycolysis, a phenomenon known as the “Warburg effect” [3–5]. However, increased glycolysis alone is insufficient to meet the total metabolic demands of proliferating cancer cells. Elevated glutaminolysis is another hallmark of cancer [6–11].

Glutamate production by mitochondrial glutaminase, the first enzyme in glutaminolysis, is a key process for glutamine-dependent anapleurosis and glutathione biosynthesis [12]. There are two predominant human isozymes of glutaminase, GLS1 and GLS2, which exhibit distinct tissue distributions and are regulated quite differently [13, 14]. It has been shown that the Myc family member, c-Myc, indirectly stimulates GLS1 expression in P493 Burkitt's lymphoma and PC3 prostate cancer cells through suppression of miR-23a/b [15]. In sharp contrast, both p53 and p63 tumor suppressors were shown to specifically activate GLS2 to support cellular defense against oxidative stress and oncogenic transformation [16–18]. It thus appears that GLS1 and GLS2 execute opposite functions in malignant transformation. In support of this notion, GLS1 expression is markedly elevated whereas GLS2 expression is decreased in hepatocellular carcinoma relative to normal liver tissues [19], and ectopic GLS2 expression reduced colony formation *in vitro* [16, 17].

However, given the incredible genetic and microenvironmental diversities across cancer types, do cancer cells exclusively upregulate GLS1 while downregulate GLS2 to sustain glutaminolysis and TCA cycle replenishment? More importantly, another essential Myc family member, N-Myc, also similarly potentiates GLS1 activation to engage glutamine-dependent anapleurosis? In this regard, *MYCN*-amplified neuroblastomas provide an attractive model for studying the molecular mechanisms that underlie the connection between N-Myc overexpression and glutamine addiction [20, 21]. We therefore sought to address these important questions in *MYCN*-amplified neuroblastoma cells and primary tumors.

## RESULTS

### N-Myc promotes GLS2 activation in *MYCN*-amplified neuroblastoma cells

**Figure 1:**
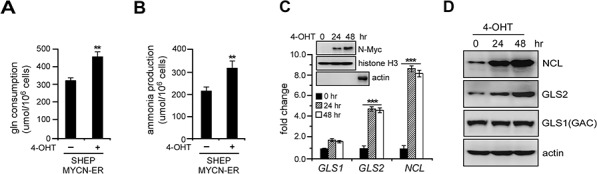
N-Myc induction promotes glutamine catabolism in association with GLS2 activation **A.** and **B.** N-Myc activation promotes glutamine metabolism. SHEP MYCN-ER cells were treated with or without 100 nM 4-hydroxytamoxifen (4-OHT) for 24 hrs. Glutamine (A) and ammonium (B) levels in the media were analyzed using the Nova Flex and are presented as an average of triplicates. **C–D.** MYCN-ER induction activates GLS2 but not GLS1 expression. SHEP MYCN-ER cells were treated with 4-OHT for 0, 24 and 48 hrs. GLS1 and GLS2 levels were quantitated by real-time qPCR using ΔCT method (C) and immunoblot assays (D) *NCL*, which encodes nucleolin, was used as a positive control to monitor Myc transcriptional activities. Data shown are averages of representative triplicates from one cDNA sample. Actin was used as a loading control. Inset: nuclear fractions were isolated from SHEP MYCN-ER cells treated by 4-OHT as indicated, and N-Myc nuclear translocation was monitored by immunoblot. Histone H3 was used as a nuclear marker (also a loading control) and actin as a cytoplasmic marker (the last band in actin immunoblot was obtained from total cell extracts to confirm the efficacy of actin antibody). **p < 0.01; ***p < 0.005.

To evaluate the impact of N-Myc on oxidative glutamine metabolism, we analyzed glutamine consumption and ammonia production in SHEP MYCN-ER, a *MYCN* single-copy neuroblastoma cell line bearing a 4-hydroxytamoxifen (4-OHT) activating *MYCN* transgene. As expected, administration of 4-OHT in SHEP MYCN-ER cells led to a significant increase in glutamine consumption and ammonia production (Figures 1A and 1B). We then examined glutaminase expression upon N-Myc activation. Surprisingly, MYCN-ER induction caused a time-dependent activation of GLS2 and nucleolin (a well-known N-Myc target encoded by *NCL* gene) expression without appreciable effect on that of GLS1 (Figures 1C and 1D), suggesting that N-Myc promotes selective GLS2 but not GLS1 induction in this context. Human GLS1 contains two isoforms, KGA (kidney-type glutaminase, molecular weight ~72 KD) and GAC (glutaminase C, molecular weight ~53 KD), which are formed by alternative splicing of the same mRNA transcript [22]. Using an antibody recognizing both isoforms of GLS1, we only detected the 53 KD protein band of GAC isoform in neuroblastoma cell lysates (Figure 1D), which was further confirmed by shRNA depletion in additional neuroblastoma cell lines (Supplementary Figure S1), demonstrating that GAC is the predominant GLS1 isoform expressed in human neuroblastoma cells.

**Figure 2:**
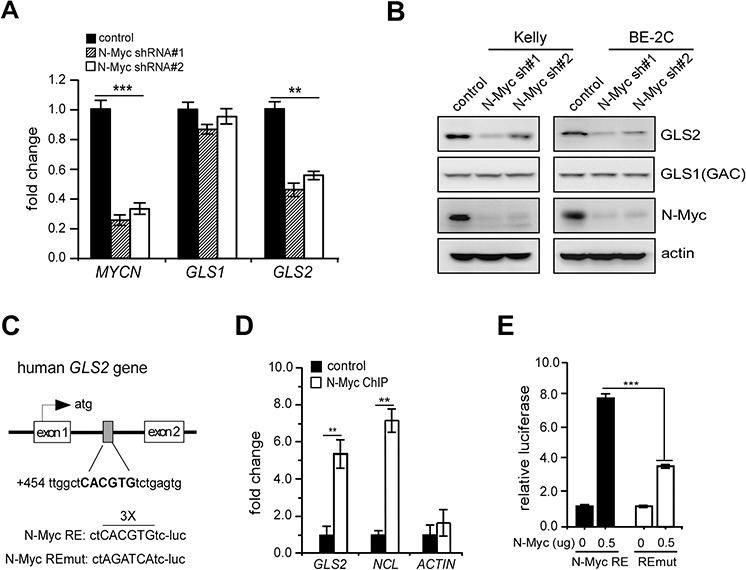
N-Myc is a novel GLS2 activator **A.** and **B.** Effect of N-Myc depletion on GLS1 and GLS2 expression in Kelly and BE-2C cells. Relative *MYCN*, *GLS1* and *GLS2* mRNA levels were quantitated by real-time qPCR by the ΔCT quantitation method (A); Data shown are averages of representative triplicates from one cDNA sample. Relative protein levels were quantitated by western blot (B); actin was used as a loading control. **C.** Schematic representation of the Myc response element (Myc RE) within the first intron of *GLS2* and its mutant (REmut). **D.** Binding of N-Myc to the *GLS2*, *NCL* and *ACTIN* promoters analyzed by ChIP assay in Kelly cells with a specific N-Myc antibody or isotype control IgG. Results are presented as averages of fold difference between N-Myc ChIP and IgG control (background) in triplicates. **E.** Luciferase assay performed using Myc RE and REmut constructs in the presence or absence of exogenous N-Myc expression. Data shown are averages of triplicates. **p < 0.01; ***p < 0.005.

To directly evaluate its roles in this event, we depleted N-Myc expression by two specific shRNAs in Kelly and BE-2C, two *MYCN*-amplified neuroblastoma cell lines exclusively expressing high levels of N-Myc protein (Supplementary Figure S2). Consistently, N-Myc depletion caused a significant decrease in GLS2 but not GLS1 expression in either Kelly or BE-2C cells (Figures 2A and 2B). Consistent with a previous study [15], we confirmed that c-Myc inactivation in P493 cells caused a marked decline in expression of 53 KD GAC (Supplementary Figure S3), the GLS1 isoform predominantly expressed in these cells [23]. These results reinforce the notion that specific target gene promoters and cancer cell types act in concert to determine Myc (c-Myc or N-Myc) transcriptional activities [24]. Surprisingly, p53 inhibition had no effect on GLS2 induction in neuroblastoma cells, whereas the expression of p21, another p53 target involved in cell cycle progression, was inhibited under the same conditions (Supplementary Figure S4). Altogether, these data suggest that N-Myc promotes oxidative glutamine metabolism predominantly through selective GLS2 induction in *MYCN*-amplified neuroblastoma cells.

We next performed in silico analysis and identified a canonical Myc binding site within the first intron of *GLS2* (Figure 2C). Chromatin immunoprecipitation (ChIP) assay revealed a significant increase in N-Myc recruitment to the first intron of *GLS2* gene when compared with the IgG control (Figure 2D). Nucleolin and actin promoters were used as positive and negative controls (Figure 2D). We then created luciferase reporter constructs using a pGL3 plasmid containing the putative Myc binding site or its mutant (Myc RE-luc and REmut-luc, Figure 2C). As expected, ectopic N-Myc expression significantly activated the wild-type Myc-RE luciferase activity when compared with REmut-luc (Figure 2E). Although exogenous c-Myc similarly activates this reporter in 293T cells (Supplementary Figure S5), it is unlikely to contribute to GLS2 activation in *MYCN*-amplified neuroblastoma cells as these cells barely exhibit detec-Myc expression (Supplementary Figure S2). Taken together, these results support that N-Myc selectively activates GLS2 expression to promote glutamine catabolism in *MYCN*-amplified neuroblastoma cells.

### GLS2 suppression inhibits the growth and survival of *MYCN*-amplified neuroblastoma cells *in vitro* and *in vivo*

**Figure 3:**
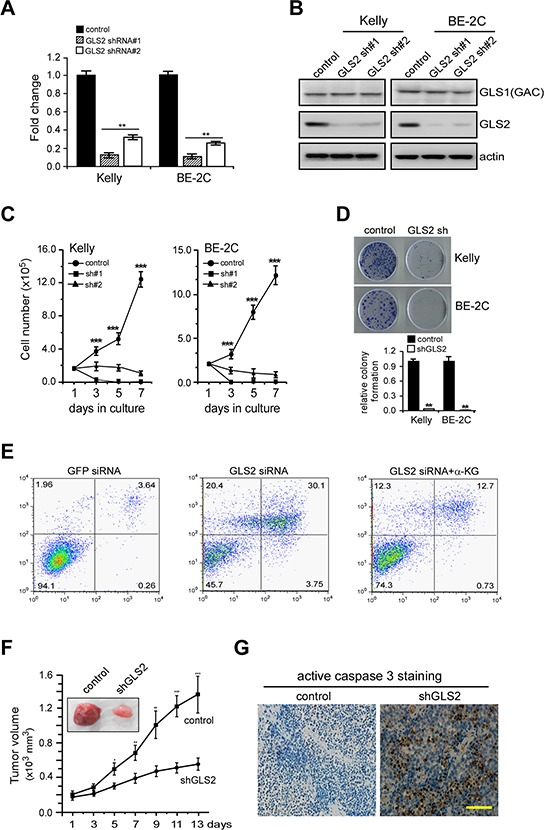
GLS2 sustains proliferation and viability of *MYCN*-Amplified neuroblastoma cells **A.** and **B.** Depletion of GLS2 expression by two specific shRNAs in Kelly and BE-2C cells. Relative *GLS2* mRNA levels were quantitated by real-time qPCR (A); data shown are an average of triplicates from a single cDNA. GLS1 (GAC) and GLS2 protein levels were analyzed by western blot (B); actin was used as a loading control. **C.** Proliferation of Kelly and BE-2C cells cultured over 7 days, as measured by serial cell counts upon GLS2 inhibition. Data are shown as an average of triplicates. **D.** Relative clonogenic growth of Kelly and BE-2C cells expressing a control or specific GLS2 shRNA. **E.** Representative PI–Annexin V staining plots of Kelly cells treated with control or GLS2 siRNA or combination of GLS2 siRNA and dimethyl α-KG (4 mM). **F.** Depletion of GLS2 expression profoundly inhibited the xenograft tumor growth of Kelly cells (*n* = 6 tumors per group). **G.** Representative staining of active caspase3 in tumor sections with and without GLS2 inhibition. The scale bar represents 50 μm. **p < 0.01; ***p < 0.005.

We then sought to determine whether elevated GLS2 expression is functionally linked to N-Myc mediated neuroblastoma cell proliferation. To directly evaluate its function in cell growth and survival, we depleted GLS2 expression in Kelly and BE-2C cells by two specific shRNAs (Figures 3A and 3B). Note that GLS2 shRNAs had no appreciable effect on GAC expression (Figure 3B). We then quantified the viable cells cultured in the presence of 2 mM glutamine over 7 days (Figure 3C). Whereas control cells substantially proliferate over time, proliferation of all the GLS2-depleted cells were pronouncedly reduced. Moreover, GLS2 depletion almost completely abrogated the clonogenic capacity of Kelly and BE-2C cells (Figure 3D). Although not activated by N-Myc (Figures 2A and 2B), GLS1 depletion also inhibited proliferation of these cells (Supplementary Figure S6). These data suggest that GLS2 might cooperate with GLS1 to promote proliferation and survival of *MYCN*-amplified neuroblastoma cells, at least *in vitro*.

We previously showed that *MYCN*-amplified neuroblastoma cells strictly rely on glutamine-dependent anapleurosis to maintain TCA cycle activity and cell viability [20, 21]. Accordingly, glutamine deprivation depletes TCA cycle intermediates and induces dramatic cell death in *MYCN*-amplified neuroblastoma cells [20]. We reasoned that if GLS2-mediated glutamine deamidation is critical to the survival of *MYCN*-amplified neuroblastoma cells, then, depletion of GLS2 expression should achieve a phenotype similar to that caused by glutamine starvation. As expected, inhibition of GLS2 expression in Kelly cells resulted in profound apoptosis similarly as glutamine deprivation (Figures 3E and Supplementary Figure S7). More strikingly, addition of dimethyl α-ketoglutarate (α-KG), a cell-permeable α-KG analog, significantly suppressed apoptosis of Kelly cells induced by GLS2 knockdown (Figure 3E), suggesting that *MYCN*-amplified neuroblastoma cells rely on GLS2-mediated glutaminolysis to replenish TCA cycle intermediates essential for mitochondrial integrity and cell survival.

We then established subcutaneous xenografts using Kelly cells to confirm whether GLS2-mediated glutamine catabolism could affect the tumorigenic capacity of *MYCN*-amplified neuroblastoma cells *in vivo*. We generated Kelly cells expressing a control or GLS2-specific shRNA. Strikingly, GLS2 knockdown markedly attenuated the ability of Kelly cells to form tumors *in vivo* (Figure 3F). Consistent with the results obtained *in vitro* (Figures 3C–3E), depletion of GLS2 activity induced massive cell death *in vivo* (Figure 3G). Taken together, these results demonstrate an important role of GLS2 in oxidative glutamine metabolism driven by oncogenic N-Myc, suggesting targeting GLS2 may represent an effective treatment approach to neuroblastoma patients exhibiting *MYCN*-amplification.

### GLS2 depletion inhibits both glutamine-dependent anapleurosis and aerobic glycolysis

**Figure 4:**
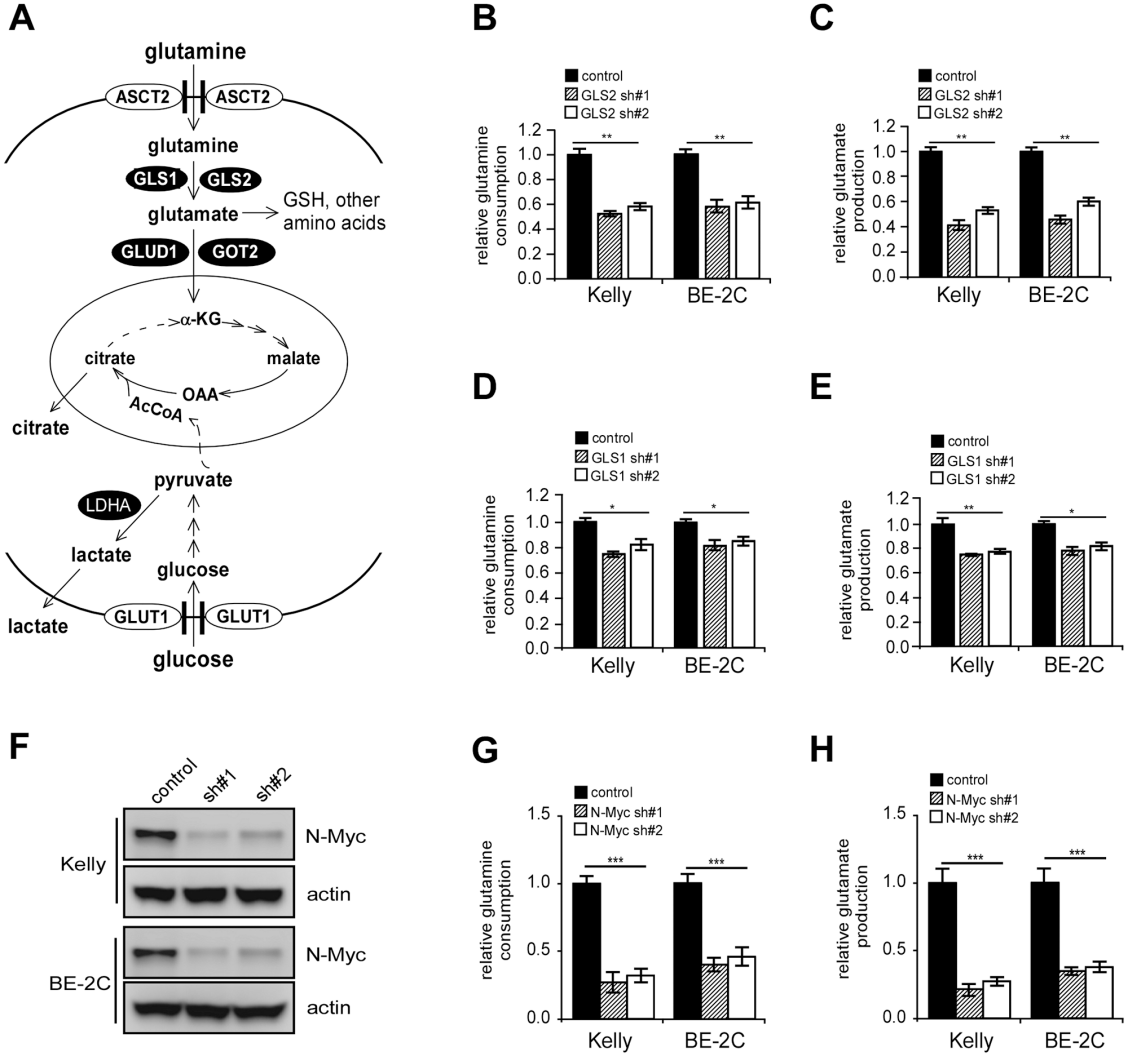
GLS2 depletion inhibits conversion of glutamine to glutamate **A.** Diagram depicting glutamine and glucose metabolism. See text for more details. **B.** and **C.** Effect of GLS2 inhibition on glutamine consumption (B) and glutamate production (C) **D.** and **E.** Effect of GLS1 depletion on glutamine consumption (D) and glutamate production (E) analyzed as in (B) and (C). **F–H.** Effect of N-Myc depletion (F) on glutamine consumption (G) and glutamate production (H) analyzed as in (B) and (C) Kelly or BE-2C cells were infected with indicated shRNAs and selected with puromycin for 24 hr, and then switched to fresh medium. After 24 hr, glutamine consumption and glutamate production was analyzed by respective assay kits, and normalized to the same cell number. Data were presented as percentages of control and are shown as averages of triplicates. *p < 0.05; **p < 0.01; ***p < 0.005.

Glutamine is converted by GLS1 and GLS2 to glutamate for either glutathione (GSH) biosynthesis or further catabolism by the TCA cycle (Figure 4A). To evaluate GLS1 and GLS2 in oxidative glutamine metabolism, we analyzed glutamine consumption and glutamate production in Kelly and BE-2C cells. As expected, GLS2 depletion significantly inhibited glutamine consumption and subsequent glutamate production (Figures 4B and 4C), while downregulation of GLS1 expression resulted in a weaker inhibition (Figures 4D and 4E), suggesting a dominant role of GLS2 in this event. Notably, abrogation of N-Myc function also significantly inhibited glutamine consumption and glutamate production (Figures 4F–4H), arguing that N-Myc promotes glutamine deamidation in *MYCN*-amplified neuroblastoma cells in part through GLS2 activation.

**Figure 5:**
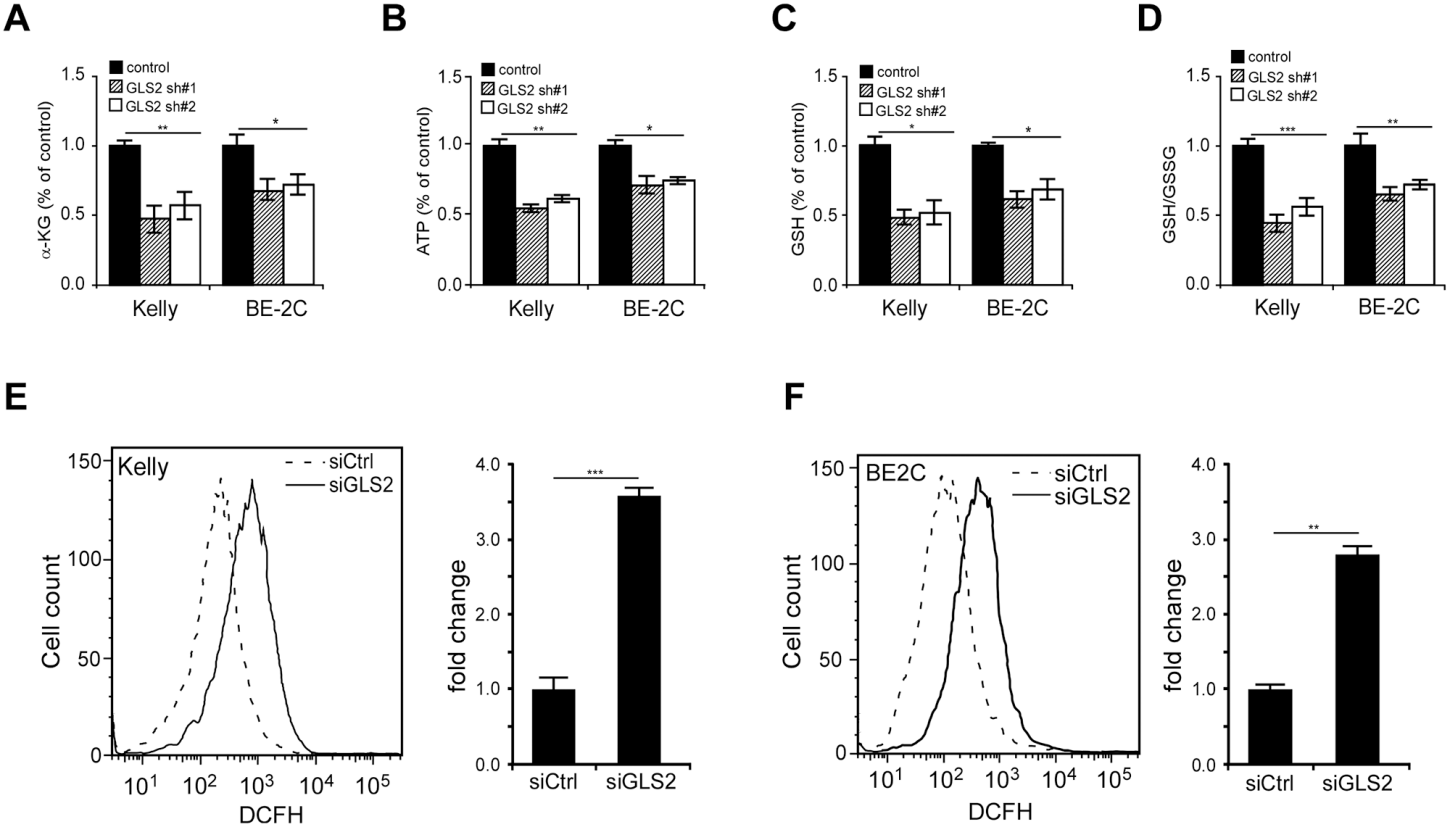
Changes in α-KG contents, ATP production, GSH biosynthesis and ROS generation upon GLS2 depletion **A–D.** Effects of GLS2 depleted on contents of α-KG (A), ATP (B) and GSH (C), as well as GSH/GSSG ratio (D). Kelly and BE-2C cells were infected with indicated GLS2 shRNAs and selected with puromycin for 24 hr, and then switched to fresh medium. After 24 hr, α-KG, ATP, and GSH contents were analyzed with respective assay kits and normalized to the same cell number. Data were presented as percentages of control and are shown as averages of triplicates. **E–F.** Effects of GLS2 depletion on ROS production in Kelly (E) and BE-2C (F) cells. Cells were transfected with indicated siRNAs for 40 hr. DCF staining was followed by FACS analysis. Fold changes in ROS generation were presented as an average of triplicates. *p < 0.05; **p < 0.01; ***p < 0.005.

To examine whether GLS2-mediated glutamine oxidation sustains TCA cycle progression, we analyzed the abundance of α-KG, a critical metabolite immediately downstream of glutamate, and found that GLS2 elimination dramatically decreased the intracellular α-KG levels in Kelly cells by approximately 50% (Figure 5A). Further analysis showed that inhibition of GLS2 expression also profoundly inhibited ATP generation (Figure 5B), validating our previous findings that glutamine metabolism provides an important energy source (ATP) to support neuroblastoma cell growth [20, 21].

Glutamate is a precursor of GSH, a critical antioxidant molecule and a scavenger for reactive oxygen species (ROS). Once electrons get lost, the GSH becomes oxidized (GSSG). Interestingly, GLS2 knockdown significantly decreased both GSH content and GSH/GSSG ratio (Figures 5C and 5D), suggesting that enhanced glutamine deamidation by GLS2 plays an important role in proper redox homeostasis maintenance in *MYCN*-amplified neuroblastoma cells. In support of this notion, siRNA knockdown of GLS2 expression profoundly increased ROS levels in both Kelly and BE-2C cells in comparison with the mock counterparts (Figures 5E and 5F). As a further support of N-Myc's critical roles in promotion of glutamine-dependent anapleurosis, its depletion also significantly decreased the abundance of α-KG, ATP, and GSH (Supplementary Figure S8).

**Figure 6:**
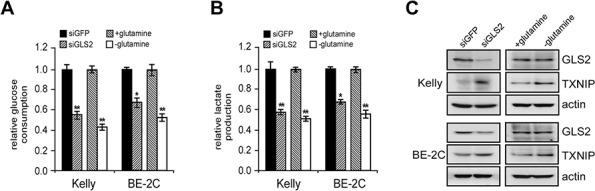
GLS2 depletion inhibits aerobic glycolysis Glucose consumption **A.** and lactate production **B.** upon GLS2 inhibition or glutamine deprivation. Kelly and BE-2C cells were transfected with indicated siRNAs for 24 hr and then switched to fresh medium. 24 hr later, glucose consumption and lactate production were analyzed with respective assay kits and normalized to the same cell number. Kelly and BE-2C cells that were glutamine-starved for 24 hr were used for comparison. Data were presented as percentages of control and are shown as averages of triplicates. **C.** GLS2 and TXNIP protein levels analyzed by western blot. Actin was used as a loading control. *p < 0.05; **p < 0.01.

A previous study showed that glutamine-dependent anapleurosis dictates glucose uptake and cell growth in some transformed cell lines by suppression of TXNIP (a negative regulator of glucose uptake) induction [25]. Interestingly, we noticed glutamine deprivation similarly resulted in TXNIP induction and subsequent decrease in glucose uptake and lactate secretion in *MYCN*-amplified neuroblastoma cells (Figures 6A–6C). We reasoned if GLS2 activity is indeed essential for glutamine-dependent anapleurosis in *MYCN*-amplified neuroblastoma cells, then, GLS2 suppression should recapitulate glutamine deprivation in regulation of glucose metabolism. As expected, like glutamine starvation, abrogation of GLS2 expression induced a similar TXNIP activation concomitant with a dramatic decline in both glucose uptake and lactate production (Figures 6A–6C). Whether TXNIP virtually functions downstream of GLS2 to restrict aerobic glycolysis needs further investigation, though we observed prominent induction of this protein in *MYCN*-amplified neuroblastoma cells upon glutamine starvation and GLS2 knockdown. Nevertheless, these data demonstrated that GLS2-mediated glutamine catabolism coordinates both of the energy-generating pathways, oxidative phosphorylation and aerobic glycolysis, to stimulate neuroblastoma cell growth and proliferation. These results also suggest that, in addition to inhibition of glutamine metabolism, a loss of aerobic glycolysis might also contribute to decreased production of α-KG, ATP and other critical metabolites upon GLS2 inhibition.

### GLS2 expression is significantly elevated in *MYCN*-amplified neuroblastomas and represents a potential biomarker for patient prognosis

**Figure 7:**
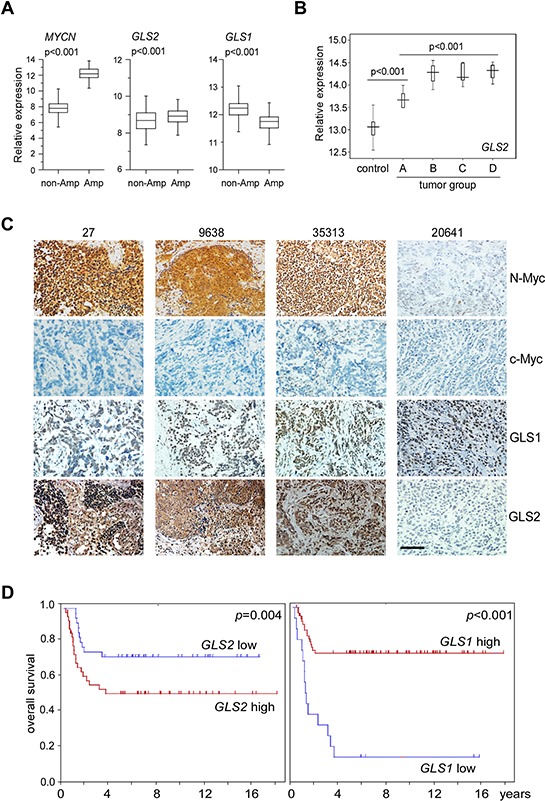
Expression of GLS1 and GLS2 in primary neuroblastoma tumors **A.** Relative expression of *MYCN*, *GLS1* and *GLS2* in 643 human neuroblastoma tumors. Non-Amp: *MYCN* non-amplified tumors (550); Amp: *MYCN*-amplified tumors (93). **B.** Relative *GLS2* expression in *MYCN*-transgenic mouse neuroblastoma tumors. Control: mouse sympathetic ganglia; A, B, C and D represent tumor groups with an increasing malignancy, respectively. **C.** Representative N-Myc, c-Myc, GLS1 and GLS2 immunochemical staining in *MYCN*-amplified neuroblastoma tumors; sections from 20641 (a *MYCN* non-amplified, low-stage neuroblastoma tumor) were used as a negative control. The scale bar represents 50 μm. **D.** Kaplan–Meier survival curvesn of neuroblastoma patients based on *GLS1* and *GLS2* expression. Data were generated from Kocak dataset accessible at http://r2.amc.nl.

To confirm that our findings in cell lines resemble that of *MYCN*-amplified neuroblastoma patient populations, we analyzed the microarray data obtained from 643 primary neuroblastoma tumors. In support of its predominant role in controlling glutamine deamidation in *MYCN*-amplified cell lines, *GLS2* expression is significantly elevated in the *MYCN*-amplified group when compared with non-amplified tumors, while expression of *GLS1* is surprisingly reduced (Figure 7A). Analysis of microarray data [26] obtained from mouse neuroblastoma tumors bearing the human *MYCN* transgene further corroborated that *GLS2* expression was significantly elevated during aggressive tumor progression (Figure 7B). The *GLS1* probe was not included, thus preventing further evaluation of its expression in this tumor dataset. Subsequent immunochemistry staining confirmed that expression of GLS2, but not GLS1, was markedly elevated in *MYCN*-amplified tumors (samples 27, 9638 and 35313) when compared with the low-stage, non-amplified tumor (sample 20641, Figure 7C). Note that these examined neuroblastoma tumors exhibited undetec-Myc expression (Figure 7C). The observation that N-Myc dependent activation of GLS2 expression also prompted us to assess its potential prognostic significance in human patients. Indeed, elevated *GLS2* expression is significantly correlated with a poor neuroblastoma patient survival (Figure 7D). Paradoxically, *GLS1* expression was negatively associated with prognosis of these individuals (Figure 7D). Taken together, these results suggest that GLS1 vs GLS2 status might be used as a potential predictor in neuroblastoma patient diagnosis.

## DISCUSSION

Both c-Myc and N-Myc regulate multiple aspects of tumor metabolism, enabling cancer cells to avidly uptake both glucose and glutamine [27]. Our results reveal that metabolic control of N-Myc induced neuroblastomas differs from that of c-Myc transformed Burkitt's lymphomas, with one of the checkpoints being differential regulation of GLS1 versus GLS2 activation. More importantly, in contrast with its tumor suppressive roles in hepatocellular carcinomas [16, 17, 28], we showed that GLS2 paradoxically coordinates both glutamine-dependent anapleurosis and aerobic glycolysis to sustain cell proliferation and survival of *MYCN*-amplified neuroblastomas. Of note, GLS2 is substantially upregulated in human neuroblastoma tumors harboring *MYCN* amplification, and correlates with increased malignancy in a mouse transgenic neuroblastoma model. Moreover, in examination of GLS2 expression in 144 cases of human cervical cancer specimens (58 radioresistant samples+86 radiosensitive samples) and 15 adjacent normal tissues, Xiang and colleagues identified GLS2 expression was significantly elevated in tumor specimens obtained from radioresistant patients [29]. In particular, abrogation of GLS2 expression recovered radiosensitivities of resistant tumor cells, *in vitro* and *vivo*, concomitant with a significant increase in ROS production [29]. Thus, at least in neuroblastomas and cervical carcinomas, GLS2 appears to promoting an opposite phenotype even though it facilitates the similar metabolic effects as those observed in hepatocellular carcinoma cells. The exact mechanisms involved in regulation of the tumor-promoting versus tumor-suppressing functions of GLS2 are currently unclear. Context-dependent GLS2 regulation may account for the divergent phenotypes observed. Additionally, differential metabolic requirements within specific cancer types might contribute to dictating the final outcome of deregulated GLS2 activities. In support of this notion, we previously demonstrated that, unlike hepatocellular carcinoma cells, *MYCN*-amplified neuroblastomas strictly rely on large amounts of exogenous glutamine for cell survival [20, 21]. Here we further showed that N-Myc promotes glutaminolysis through selective activation of GLS2 expression. Conceivably, a consequence of this N-Myc dependent GLS2 activation is the reprogramming of mitochondrial metabolism to depend on glutamine catabolism to sustain cellular viability and TCA cycle anapleurosis, triggering neuroblastoma cellular addiction to glutamine as a bioenergetic substrate. These results also revise traditional view of GLS2 function and suggest that it might act as a double-edged sword in regulating cellular activities, depending on upstream signals and enforced metabolic dependencies.

Proliferation and metabolism are precisely coordinated processes during normal cell growth and division and are frequently deregulated in cancer. Despite a growing appreciation that elevated glutamine metabolism is a unifying feature of cancer [12], it remains largely unclear whether cancer cells of different origins use common or unique signaling pathways to regulate glutamine catabolism. GLS1 and GLS2 are regulated quite differently. Multiple signaling pathways, including c-Myc, NF-κB and RAF, activate GLS1 induction in human cancer cells [15, 23, 30]. In comparison, function and regulation of GLS2 is still under exploration, although it was recently shown to be linked to p53 or p63 pathway as a tumor suppressor when ectopically overexpressed [16–18].

Unexpectedly, we showed in the current study that *MYCN*-amplified neuroblastoma cells predominantly rely on activation of GLS2-mediated glutamine deamidation to sustain TCA cycle anapleurosis and biosynthetic activities. Importantly, we uncovered a previously unknown mechanism involved in regulation of GLS2 expression in *MYCN*-amplified neuroblastoma cells, and identified N-Myc as a novel activator selectively upregulating GLS2 (but not GLS1) expression. Surprisingly, while elevated GLS2 expression is significantly correlated with a poor neuroblastoma patient survival, GLS1 expression was paradoxically associated with favorable prognosis in these patient populations. Why would two proteins that perform identical functions (both enzymes catalyze glutamine deamidation, the first rate-limiting step in glutamine catabolism) exhibit opposing correlations as to overall neuroblastoma patient survival? Differential cellular localizations of GLS1 and GLS2 might account for their possibly distinct functions observed in human patients. While the KGA isoform of GLS1 is localized in the cytoplasm, GLS2 is predominantly distributed in mitochondria. Presumably, N-Myc dependent activation of mitochondrial GLS2 might provide for the increased metabolic and biosynthetic needs, thus conferring selective advantages to aggressive neuroblastoma progression. However, our *in vitro* studies showed that neuroblastoma cells exhibited undetectable KGA expression; instead, these cells predominantly express GAC, the GLS1 isoform primarily localized in the mitochondria. These results suggest that cellular localizations per se can hardly explain the distinct effects of GLS1 versus GLS2 in neuroblastoma patient survival. It is also likely that alternate activities of these enzymes could play a role in their divergent effects. Unlike GAC, GLS2 is predicted to contain an ankyrin-repeat domain in its C-terminal region [31], which may impart differential properties and functions to GLS2 through protein-protein interactions [28, 32]. Nevertheless, results shown here depicted a new layer of regulation in control of tumor glutamine metabolism and provided a molecular explanation for the functional significance of GLS2 overexpression observed in *MYCN*-amplified neuroblastomas.

Taken together, these results reinforced the notion that a unified model of altered tumor metabolism might not exist. Instead, the diversities within metabolic programs of specific cancer cells can dictate by what means the proliferative rewiring is fueled, which in turn imparts heterogeneities of metabolic dependencies of these cells. Thus, a better understanding of these metabolic diversities will improve our ability to define their contribution to aggressive tumor progression. In aggregate, our findings not only identify a potentially useful biomarker for neuroblastoma patient stratification but also provide a mechanistic basis for differences in glutamine metabolism, a key driver of tumor development and response to therapy.

## MATERIALS AND METHODS

### Cell culture

All the neuroblastoma cell lines were kindly provided by Drs. John M Maris and Michael D Hogarty at Children's Hospital of Philadelphia, University of Pennsylvania, USA. Neuroblastoma cells were maintained in RPMI media containing 10% FBS and 2 mM glutamine. All the cells used in the experiments were validated as Mycoplasma-negative.

### RNA interference

Specific shRNAs against N-Myc, p53, GLS1, GLS2 or GFP were obtained from Sigma. After viral transduction, cells were selected with puromycin (Sigma). GLS2 and control siRNAs were obtained from Qiagen. The silencing efficacy of respective shRNAs or siRNAs was confirmed by QRT-PCR and Western blot.

### QRT-PCR

Total RNA was extracted with Trizol reagent following the manufacturer's instructions (Invitrogen). The relative levels of respective genes were examined using specific Taqman primers (Applied Biosystems) and normalized in reference to actin expression and presented as a percentage of the control.

### Metabolic analysis

Glutamine and ammonium levels in the medium were analyzed using the Nova Flex. Glucose, lactate, glutamate, α-KG and GSH levels were determined using respective assay kits purchased from BioVision. Data are an average of triplicates and presented as a percentage of the control group.

### ROS detection

Kelly and BE-2C cells were incubated with 2′, 7′-dichlorodihydrofluorescein diacetate (DCF; Molecular Probes) for 10 min at 37°C. After incubation, cells were washed with PBS, trypsinized and resuspended in PBS solution. Fluorescence was measured using a FACS can flow cytometer and data analyzed with CELL Quest software.

### Cell death assay

Cells were harvested by combining floating cells in the medium and adherent cells detached by trypsin, and cell pellets were washed once with cold PBS. Apoptosis was analyzed by the Annexin V-FITC Apoptosis Kit (BioVision).

### Luciferase reporter assay

pGL3 expressing Myc-RE or indicated mutant was transiently cotransfected in triplicates into 293T cells using Fugene 6 with Renilla luciferase reporter. When indicated, pCMV-N-Myc plasmid was included. Luciferase activities were measured 16–20 hr later with a Dual Luciferase Kit (Promega). Firefly luciferase activities were normalized to Renilla luciferase control values and shown as an average of triplicates.

### ChIP and western blot analysis

ChIP was performed following standard protocol from Upstate Biotech. For Western blots, cells were lysed in RIPA and 50 μg total cellular proteins were used for each blot. Antibodies were used as follows: N-Myc (Santa Cruz, sc53993), c-Myc (Santa Cruz, sc-42), GLS1 (Abcam, ab93434), GLS2 (Thermo Scientific, PA5-13602), p53 (Santa Cruz, sc-126), TXNIP (Medical and Biological Laboratories, K02053), caspase 3 (Cell Signaling, 9661) and actin (Sigma, AC-15).

### Xenograft tumors

NOD-SCID mice were injected s.c. with two million Kelly cells (control or GLS2 shRNA) diluted in 200 μL PBS containing 50% matrigel (BD Bioscience). Tumor weight was measured at the time of sacrifice. All animal experiments were performed following the university laboratory animal guidelines and with approval from the Animal Experimentations Ethics Committee of Tongji Medical College.

### Immunohistochemistry

Sections of primary neuroblastoma tumors were obtained from the Pathology Core of Tongji Hospital with informed consent and approval (number: [2014]IEC(S013)) from the Clinical Research Ethics Committee of Tongji Medical School. The procedures involving human subjects were in accordance with the Helsinki Declaration. Tumor sections were incubated with the antibodies against control IgG, N-Myc, and GLS2 overnight at 4°C. The remaining steps were performed using the DAKO CSA kit.

### Statistical analysis

Data were expressed as mean ± SD from at least three independent experiments. Relative gene expression in human (Kocak dataset, http://r2.amc.nl) and mouse (GSE17740) neuroblastoma tumor microarrays was analyzed by SPSS-18 using Independent-Samples *t*-test. Kaplan-Meier curves were estimated and compared between groups by log-rank test. All the remaining significance analyses were performed using two-tailed Student's *t*-test.

## SUPPLEMENTARY FIGURES


