# Supplementary material for: Myc promotes glutaminolysis in human neuroblastoma through direct activation of glutaminase 2
Source: Oncotarget. 2015 Oct 19;6(38):40655–66. doi: 10.18632/oncotarget.5821 (PMC4747359; doi:10.18632/oncotarget.5821)
Supplement: Supplementary file 1 [file oncotarget-06-40655-s001.pdf]

SUPPLEMENTARY FIGURES

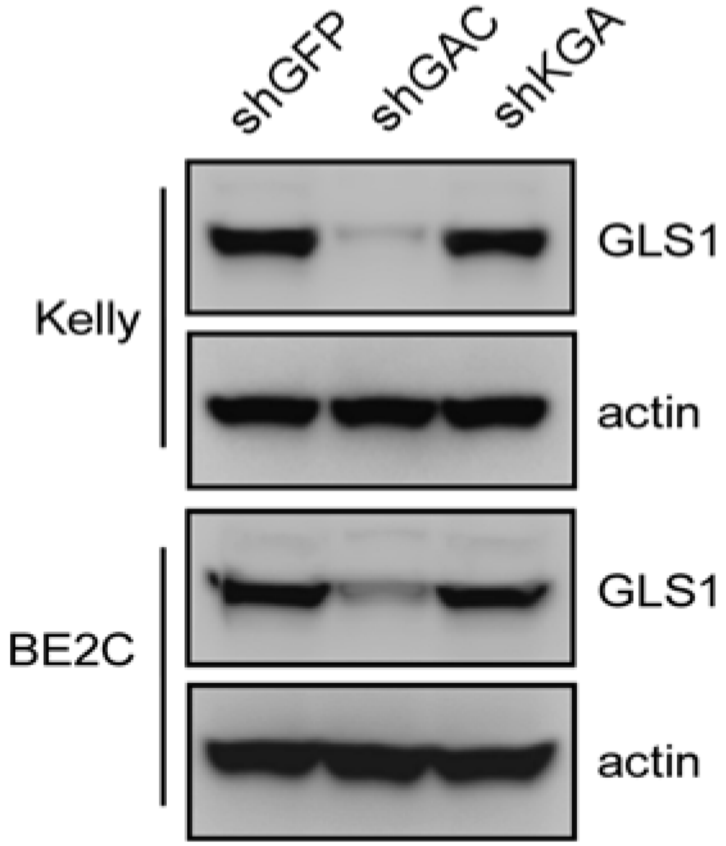

Supplementary Figure S1: *MYCN*-amplified neuroblastoma cells predominantly express GAC isoform of GLS1. KGA and GAC isoforms of GLS1 were respectively depleted by specific shRNAs in Kelly and BE-2C cells. Relative protein levels were quantitated by western blot using a commercial antibody (abcam, ab93434) recognizing both isoforms. Actin was used as a loading control.

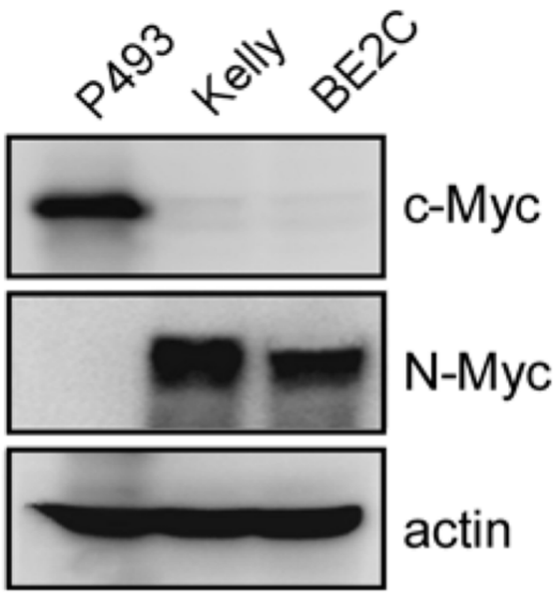

Supplementary Figure S2: Representative c-Myc and N-Myc levels in *MYCN*-amplified neuroblastoma cells. Relative protein levels were quantitated by western blot. P493 cell lysates were used as a positive control for c-Myc, and actin was used as a loading control.

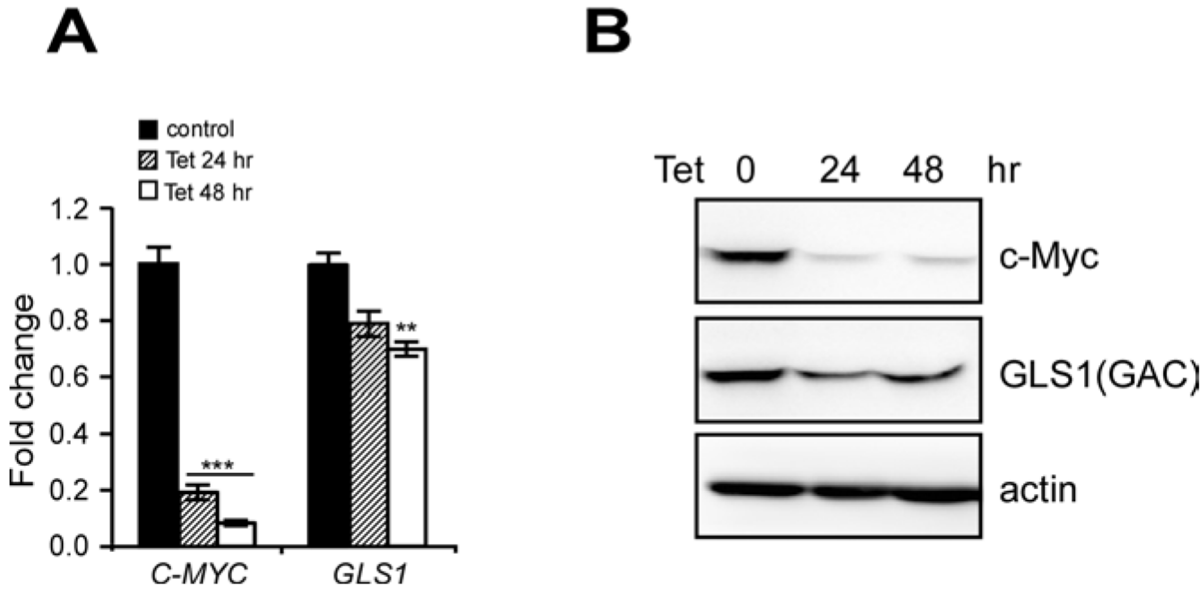

Supplementary Figure S3: c-Myc InducesGAC isoform of GLS1 in P493 Cells. Effect of c-Myc inhibition on GAC expression in P493 cells. Relative *C-MYC* and *GLS1* mRNA levels were quantitated by real-time qPCR **A.** data shown are averages of triplicates. Relative protein levels were quantitated by western blot **B.** actin was used as a loading control. ** $p < 0.01$; *** $p < 0.005$.

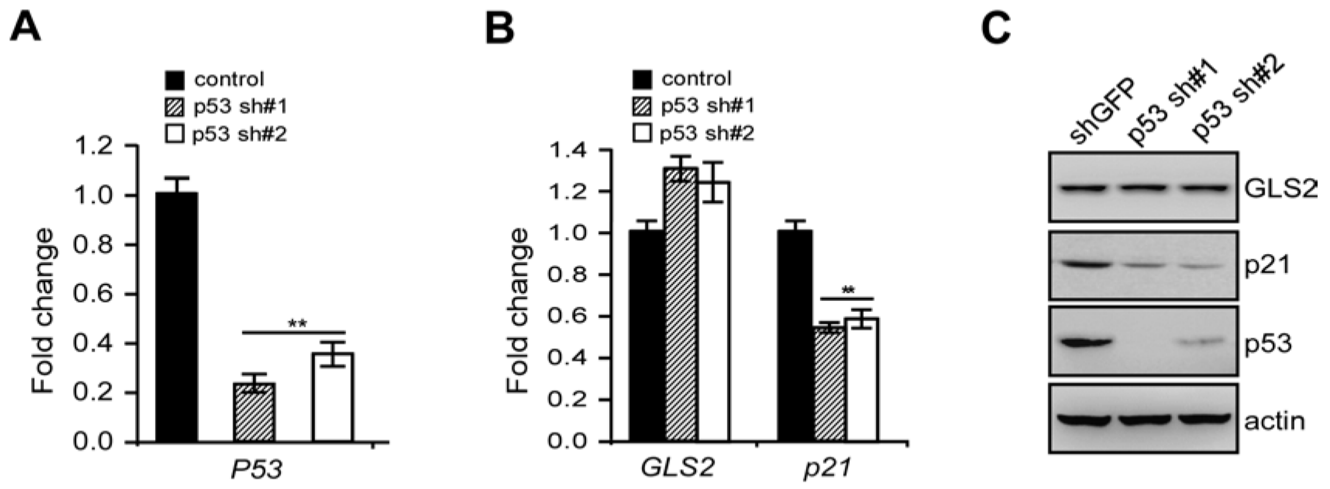

Supplementary Figure S4: P53 Does Not Affect GLS2 Expression in *MYCN*-Amplified Neuroblastoma Cells. A. Depletion of *P53* expression by two specific shRNAs in Kelly cells. Relative *P53* mRNA levels were quantitated by real-time qPCR; data shown are averages of triplicates. **B.** and **C.** Effect of p53 depletion on GLS2 and p21 expression in Kelly cells. Relative *GLS2* and *p21* mRNA levels were quantitated by real-time qPCR (B); data shown are averages of triplicates. Relative protein levels were quantitated by western blot (C); actin was used as a loading control. $^{**}p < 0.01$.

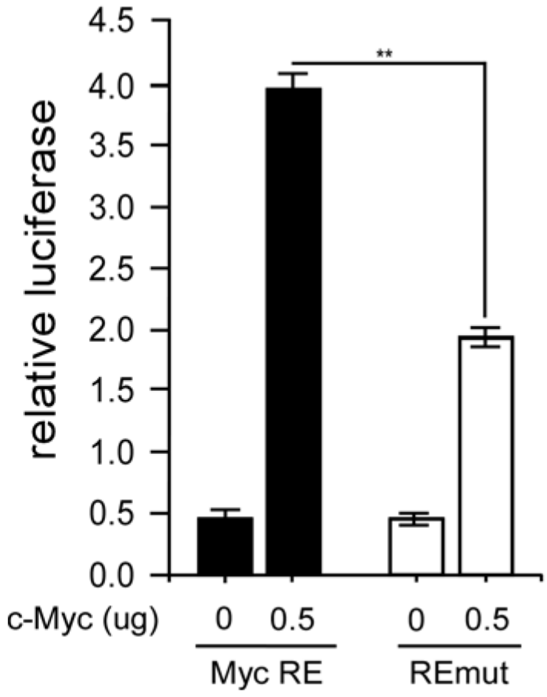

Supplementary Figure S5: Luciferase assay performed using Myc-RE and RE-mut constructs in the presence or absence of exogenous c-Myc expression. pGL3 expressing Myc-RE or indicated mutant was transiently co-transfected in triplicates into 293T cells using Fugene 6 with Renilla luciferase reporter. When indicated, pCMV-c-Myc plasmid was included. Luciferase activities were measured 16–20 hr later with a Dual Luciferase Kit. Data shown are averages of triplicates. $^{**}p < 0.01$.

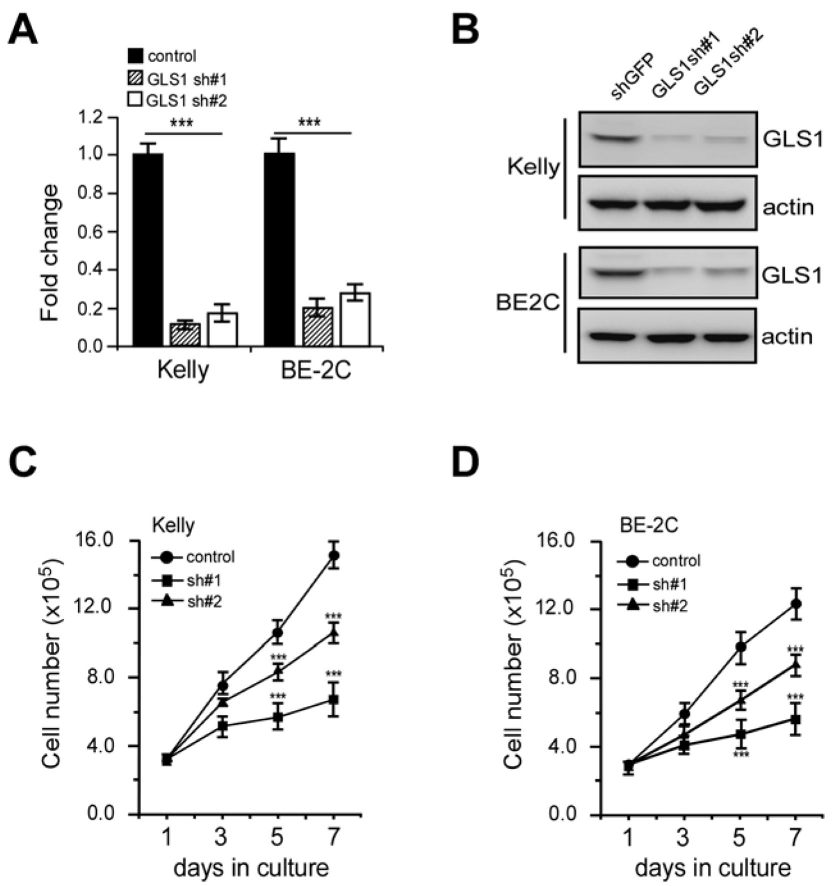

Supplementary Figure S6: GLS1 Depletion Inhibited Proliferation of Kelly and BE-2C Cells. A–B. Depletion of GLS1 expression by two specific shRNAs. Relative *GLS1* mRNA levels were quantitated by real-time qPCR (A); data shown are averages of triplicates. Relative protein levels were quantitated by western blot (B); actin was used as a loading control. ***$p < 0.005$. **C–D.** Proliferation of Kelly (C) and BE-2C (D) cells cultured over 7 days, as measured by serial cell counts upon GLS1 inhibition. Data are shown as an average of triplicates. ***$p < 0.005$.

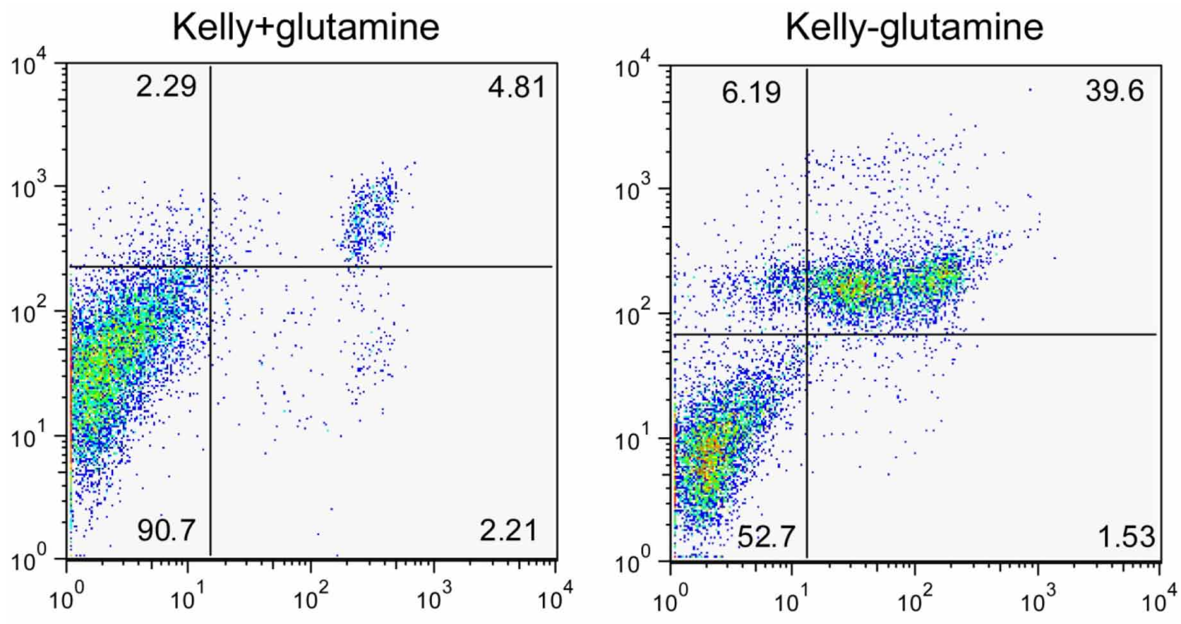

Supplementary Figure S7: Glutamine Deprivation Induced Apoptosis in *MYCN*-amplified Neuroblastoma Cells. Kelly cells were cultured in DMEM media with or without 2 mM glutamine for 48 hr, and cell viabilities were evaluated by PI–AnnexinV staining. Data shown are representative staining plots of independent triplicates.

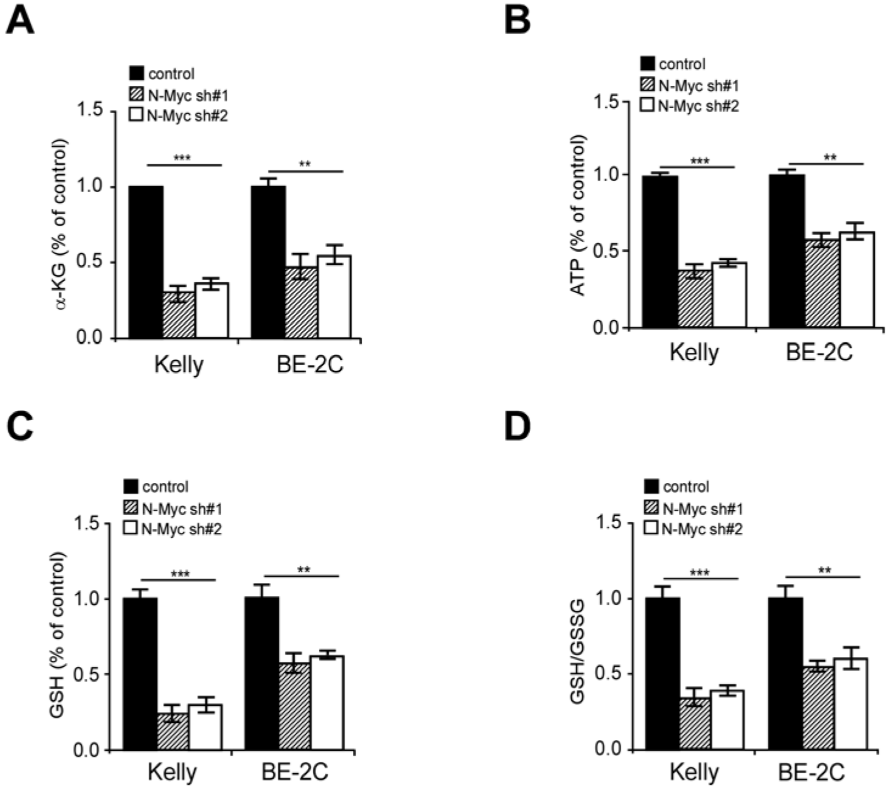

Supplementary Figure S8: N-Myc depletion inhibits α-KG generation, ATP production and GSH biosynthesis. Relative α-KG **A.** ATP **B.** GSH **C.** contents and GSH/GSSG **D.** ratio in Kelly and BE-2C cells upon N-Myc inhibition. Kelly or BE-2C cells were infected with indicated N-Myc shRNAs for 24 h and then switched to fresh medium. 24 hr later, relative α-KG, ATP, GSH contents were analyzed with respective assay kits and normalized to the same cell number. Data were presented as percentages of controls. Data shown are averages of triplicates. $^{**}p < 0.01$; $^{***}p < 0.005$.
